# The impact of an m-Health financial incentives program on the physical activity and diet of Australian truck drivers

**DOI:** 10.1186/s12889-017-4380-y

**Published:** 2017-05-18

**Authors:** Nicholas D. Gilson, Toby G Pavey, Olivia RL Wright, Corneel Vandelanotte, Mitch J Duncan, Sjaan Gomersall, Stewart G. Trost, Wendy J. Brown

**Affiliations:** 1 The University of Queensland, School of Human Movement and Nutrition Sciences, St Lucia Campus, Brisbane, Australia; 2 School of Exercise and Nutrition Sciences, Queensland University of Technology, Brisbane, Australia; 3 Central Queensland University, School for Health, Medical and Social Science, Rockhampton, Australia; 4 School of Medicine and Public Health, Priority Research Centre for Physical Activity and Nutrition, University of Newcastle, Newcastle, NSW Australia

**Keywords:** Physical activity, Diet, Small changes, m-Health intervention, Financial incentives, Truck drivers

## Abstract

**Background:**

Chronic diseases are high in truck drivers and have been linked to work routines that promote inactivity and poor diets. This feasibility study examined the extent to which an m-Health financial incentives program facilitated physical activity and healthy dietary choices in Australian truck drivers.

**Methods:**

Nineteen men (mean [SD] age = 47.5 [9.8] years; BMI = 31.2 [4.6] kg/m^2^) completed the 20-week program, and used an activity tracker and smartphone application *(Jawbone UP™)* to regulate small positive changes in occupational physical activity, and fruit, vegetable, saturated fat and processed/refined sugar food/beverage choices. Measures (baseline, end-program, 2-months follow-up; April–December 2014) were accelerometer-determined proportions of work time spent physically active, and a workday dietary questionnaire. Statistical (repeated measures ANOVA) and thematic (interviews) analyses assessed program impact.

**Results:**

Non-significant increases in the mean proportions of work time spent physically active were found at end-program and follow-up (+1%; 7 mins/day). Fruit (*p* = 0.023) and vegetable (*p* = 0.024) consumption significantly increased by one serve/day at end-program. Non-significant improvements in saturated fat (5%) and processed/refined sugar (1%) food/beverage choices were found at end-program and follow-up. Overall, 65% (*n* = 11) of drivers demonstrated positive changes in physical activity, and at least one dietary choice (e.g. saturated fat) at follow-up. Drivers found the financial incentives component of the program to be a less effective facilitator of change than the activity tracker and smartphone application, although this technology was easier to use for monitoring of physical activity than healthy dietary choices.

**Conclusions:**

Not all drivers benefitted from the program. However, positive changes for different health behaviours were observed in the majority of participants. Outcomes from this feasibility study inform future intervention development for studies with larger samples.

**Trial registration:**

ANZCTR12616001513404. Registered November 2nd, 2016 (retrospectively registered).

## Background

The truck driving industry makes major contributions to the global economy and jobs market. In Australia alone, the road transport industry contributed $73.1 billion to the national economy in 2013; reflecting trends elsewhere in the world, a 75% growth in Australian road freight is estimated over the next 20 years, with the need for 20,800 new drivers between 2013 and 2018 [1].

Set against the context of significant financial and employment contributions, and established links between poor health, fatigue, and road accidents, [2] it is cause for concern that truck drivers are an older, male dominated occupational group, characterised by a high incidence of chronic disease risk factors and conditions [3, 4]. Studies from the Americas [5, 6] and Europe [7, 8] have found long haul truck drivers to be highly susceptible to cardiovascular disease, type 2 diabetes and obesity. Our data with 44 local delivery and long haul Australian drivers (mean age 48 years) showed that although most rated their general health as good-to-excellent (73%), the majority were obese (68%) and had a high waist circumference (97%). The prevalence of systolic and diastolic hypertension was 63% and 49% respectively, and close to half (45%) were on prescribed medication or had a chronic medical condition [9].

High levels of physical inactivity and a diet high in saturated fat and processed, refined sugar are key factors underpinning the poor health status so evident in truck drivers. In a damning indictment of the industry, experts have compared truck driving to working in an ‘active living and healthy diet desert’ [3]. This is where long hours spent sitting, tight delivery deadlines, and unsupportive physical environments and work cultures restrict the time and opportunity for drivers to effectively manage energy balance through regular physical activity and healthy dietary options.

Health problems and poor lifestyle behaviours are widely recognised as being endemic to the transport industry. It is surprising therefore that only three published intervention studies have targeted physical activity and dietary improvements in truck drivers. In the first of these, researchers used lifestyle counselling to encourage physical activity and dietary changes in Finnish long distance drivers (*n* = 55) [10]. Beyond changes in weight, the study findings reported no physical activity or dietary outcomes. An individualised weight loss strategy was also the focus of the second study, whereby researchers administered a 12-week education and telephone coaching intervention to increase exercise and healthy eating in obese long distance drivers from the United States (*n* = 12) [11]. Self-reported pre-post increases in physical activity were non-significant and variable (median = +137 [IQR 369] minutes/week), but there was a decrease in saturated fat intake (median difference of -21 g). The third and final study trialled a multi-component (self-monitoring, computer training and motivational interviewing) weight loss competition targeting exercise and dietary habits in different types of drivers (national line haul, heavy haul and regional) from the United States [12]. Six months post-baseline, and relative to a control (*n* = 223), drivers in the intervention group (*n* = 229), demonstrated significant, but small increases in self-reported fruit and vegetable consumption (mean difference of 0.7 serves/day), and days of the week spent doing 30 min of moderate-to-vigorous physical activity (0.7 days/week).

Recognising this very limited evidence base and the urgent need to test scalable, practical and cost-effective intervention approaches for future industry adoption and translation, we developed a mobile-health (m-Health) financial incentives program to help truck drivers self-monitor and regulate work time physical activity and workday healthy dietary choices. The program, termed *Shifting Gears*, was tested with Queensland-based truck drivers in 2013–14 as part of the State Government’s *Healthier Happier Workplaces* initiative, and adopted an ecological, small change approach to target behaviours. This type of approach has been found to be effective and sustainable for promoting healthy lifestyles and combating energy imbalance, [13, 14] and is arguably well suited to truck driving given the time pressures, delivery deadlines and long working hours inherent in the industry.

Following on from our short report on baseline chronic disease risks, and process evaluation of smartphone use, [9] the present feasibility study reports on the main intervention outcomes for those drivers who completed the program. Consequently, the study aimed to 1) evaluate the extent to which the program facilitated changes in objectively measured work time physical activity and self-reported healthy dietary choices, and 2) qualitatively explore driver and depot manager experiences, insights and viewpoints following program completion.

## Methods

The study used a multi-method, pre-post, and follow-up design. Through existing industry contacts with two large Australian haulage companies, we sought to recruit 60 Queensland-based truck drivers from a Brisbane local delivery (*n* = 100) and long-haul (*n* = 60) depot; our target sample size was based on availability of resources for feasibility testing, with all drivers within each company alerted to the study. Interested drivers provided informed consent to participate, and were recruited through ‘toolbox’ talks, the distribution of information sheets, and individual follow-up by depot managers and Union representatives. University ethics approval was attained prior to study commencement (Approval Number 2013000750).

The intervention program (20 weeks between June–October 2014) sought to encourage increases in physical activity during driving breaks. Strategies for healthy dietary choices focused on encouraging drivers to increase workday fruit and vegetable intake, and replace foods and beverages high in saturated fat and processed, refined sugar, with lower or reduced fat and sugar options. For this feasibility study, we purposely did not engage in an overt, resource intensive educational approach, or prescribe the attainment of specific behavioural thresholds (e.g. a minimum number of daily steps). Rather we adopted a participatory, ‘light touch’ process where the drivers worked with us to share knowledge and evolve the types of small changes they considered achievable. To facilitate this process, drivers met with researchers at the depot in groups of three or four. At this briefing, we discussed, identified and mapped where positive physical activity and dietary choices could be integrated into the daily shift routine. Examples that emerged from this process included; moving rather than sitting when completing paperwork; taking a walk at a roadside rest stop, or when waiting for the truck to be loaded/unloaded at a delivery point; choosing water, rather than a soft drink, or fruit rather than chocolate or crisps during a service station break; preparing a packed lunch with fresh food, rather than buying fried food at the depot or on the road. Drivers noted their targeted small changes in a resource pack (reviewed by drivers with the researcher at the initial group briefing), and generated an action plan and personalised ‘menu’ of physical activity and healthy dietary choices for implementation across the 20-week program.

The resource pack also contained a brief introductory section describing the benefits of making small regular physical activity and healthy dietary choices, as well as user guides for an activity tracker, smartphone application, and financial incentives program. The activity tracker (provided free of charge) was the *Jawbone UP*, and as a supplement to the user guide, drivers were shown how to use this wrist-worn device, and the associated smartphone application (*UP*) to self-regulate changes following monitoring of a typical week’s behaviours. The *Jawbone UP* has been shown to have modest-to-strong agreement with accelerometer measured daily step counts, and is well-suited for participant self-monitoring in behaviour change interventions [15]. The linked financial incentives program was designed to encourage drivers to sustain engagement with the intervention and the *Jawbone UP*, by frequently logging physical activity (synchronising the activity tracker and smartphone application to upload step counts) and healthy dietary choices (manual entry or scanning a barcode).

As Table 1 describes, drivers accumulated points and received a reward relative to the number of weekly physical activity goals attained and healthy dietary choices logged, with the aim of progressing through five phases we termed *Gear Shifts*. To calculate points, researchers received permission and password-protected details from drivers to access *UP* accounts and review log history. This function summarised the number of daily step count goals achieved each week, while researchers visually tracked each day’s logs to identify healthy dietary choices from a generic list of healthy meals (e.g. fresh food instead of deep fried food), snacks (e.g. fruit instead of chocolate) and beverages (e.g. water instead of energy drinks).

**Table 1:** *Gear Shift* phases and points scheme for the incentives program

	Starting to roll with some healthy choices	20 points = $30
	Picking up speed with more healthy choices	50 points = $50
	Accelerating away from unhealthy choices	100 points = $80
	Cruising with healthy choices becoming a habit	170 points = $120
	In for the long haul with regular healthy choices	200 points = $200
Points scheme Days each week drivers achieved self-selected step goals (e.g. 1000 steps/day above a typical count established in the first week of the intervention).		
1 day = 2 points	3 days = 5 points	5 days = 10 points
Number of healthy dietary choices each week (e.g. wraps, grilled meat, sushi, salads, vegetables, fruit, nuts, water, tea, diet soft drinks or low fat milk).		
5 choices = 1 point	10 choices = 3 points	15 choices = 5 points

Using driver log data a research assistant provided individualised feedback and guidance on progress through *Gear Shifts* every 4 weeks. At 20 weeks, drivers were notified of their total points score and monetary reward (i.e. *Gear Shift* 5; 200 accumulated points; $200 voucher). Researcher support and feedback were then withdrawn, and drivers encouraged to continue monitoring and self-regulating physical activity and healthy dietary choices using the *Jawbone UP* (which they were able to keep following study completion).

Impact measures were administered at baseline (prior to driver briefings), 20 weeks on from the week each driver began implementing physical activity and dietary changes (program-end), and then again at 2-months follow-up. At these points, drivers completed a standard demographic and lifestyle survey, a battery of physical measures (height, weight, waist circumference and blood pressure), physical activity assessment, and a dietary behaviour questionnaire.

For physical activity assessment, drivers wore a GENEActiv wrist accelerometer (Activinsights Ltd., Cambridgeshire, UK) for one working week, 24 h/day (30 Hz sampling rate). This device is a tri-axial, ± 6 g seismic acceleration sensor, which is small (36x30x12 cm), lightweight (16 g), and waterproof. Using these accelerometer data, we then used a random forest activity classifier, in combination with recorded weekly shift patterns, to calculate the proportions of time drivers spent walking or running, during work time, non-work time on work days (24 h – work time), and on non-work days (24 h). As secondary outcomes of interest, the classifier was also used to assesses the proportions of sedentary (lying [sleeping] or sitting quietly) and stationary + (sitting with upper limb movement [driving] or standing) time during these periods. We have previously reported on the development and accuracy of the classifier, and highlight the prediction benefits of using this approach where the use of traditional ‘cut point’ methods are likely to misclassify sitting and driving as light/moderate intensity physical activity, compared to an algorithm that is trained to recognise arm movement in the absence of ambulation [16].

For dietary assessment, we used a brief questionnaire containing validated items measuring the number of work day fruit and vegetable serves eaten each day (two items), and the frequency of intake (never, monthly, weekly or daily) of specific types of foods (11 items; e.g. ‘how often did you eat hot chips, French fries, wedges or fried potatoes’) and beverages (18 items; e.g. ‘how often did you drink full-sugar fizzy soft drinks) [17]. Responses for food and beverage items were scored on a scale from zero (3 or more times/work day) to nine (never) - with healthy items reverse scored - then grouped to calculate a composite saturated fat (0–90) and sugar (0–68) index score (higher values indicating lower saturated fat and sugar consumption).

At end-program, we interviewed drivers and a depot manager, to capture program experiences, insights and viewpoints. Individual interviews were conducted at depots for around 20 min, recorded for analyses and identification of quotes, and adopted a semi-structured format based on the following key questions:

- In your experience, what barriers tend to limit physical activity and healthy dietary choices in drivers?
- Did the *Shifting Gears* program help you (or the drivers) become more physically active, and/or choose healthier dietary choices?
- Specifically, what did you (or the drivers), think about the activity tracker/smartphone application and the financial incentives program?
- Moving forward, what do you think the industry should be doing to encourage better driver health?

Minimal inclusion criteria for statistical analyses were 24-h accelerometer data, for at least three workdays and a non-workday, and completed dietary behaviour questionnaires, at baseline, end-program and follow-up. Data were combined for local delivery and long-haul drivers given the small number of completers in the latter group (*n* = 4). The proportions of accelerometer estimated time spent walking or running were also combined, and for ease of reference termed ‘physical activity’ in that drivers spent very little time running (on average less than 0.1% of their day). Descriptive statistics were calculated for demographics and physical measures at baseline, and for accelerometer data and dietary choices at baseline, end-program and follow-up. Repeated measures ANOVA was used to assess within driver changes (*p* < 0.05) in 1) the proportions of work time and non-work time spent physically active (and time spent sedentary and stationary+); 2) daily fruit and vegetable serves; 3) saturated fat and sugar index scores. Consistent with recognised guidelines for qualitative data analyses, [18] members of our research team (NDG, TGP) independently reviewed interview data, and then discussed the range of driver responses to agree on emergent themes and illustrative quotes relative to key questions.

## Results

A total of 44 drivers (34 local delivery and 10 long haul) provided informed consent to participate in the study (28% of drivers at both companies [*n* = 160], and a 73% recruitment rate relative to our target sample [*n* = 60]). The baseline characteristics of these drivers, along with attrition rates and reasons for dropout are described elsewhere [9]. In brief, from this number, 26 drivers started the program, and 19 drivers (15 local delivery and four long haul; 44% of the recruited sample) completed the 20 weeks.

Table 2 displays the demographic and physical characteristics of completers. These drivers were all men, who tended to be middle-aged and obese full-time or casual workers, mostly educated to diploma level. Around three-quarters did not smoke, met daily alcohol guidelines (≤2 units/day), and self-reported their health to be good or very good.

**Table 2:** Baseline demographic characteristics and physical measures (*n* = 19)

Age; mean (SD)	44.4 (10) years
Driver Type	
Local delivery	15 (79%)
Long-haul	4 (11%)
Work Status	
Full-time	9 (47%)
Casual	10 (53%)
Qualifications	
Year 10	1 (6%)
Year 12	6 (29%)
Technical or trade cert.	2 (12%)
Diploma	6 (29%)
Bachelor degree	3 (18%)
Graduate Cert	1 (6%)
Smoking Status	
Never smoked	9 (47%)
Past smoker	4 (21%)
Current smoker	6 (32%)
Alcohol Status	
Meeting daily guidelines (≤2 units/day)	13 (68%)
At risk (>2 units/day)	6 (32%)
Self-reported Health Status	
Fair	4 (24%)
Good	8 (41%)
Very good	7 (35%)
Physical measures; mean (SD)	
BMI (kg/m^2^)	31.2 (4.6)
Waist circumference (cm)	109 (10)
Systolic BP (mm Hg)	142 (12)
Diastolic BP (mm Hg)	87 (11)

*SD* standard deviation, *BP* blood pressure; all data are *n* (%) unless otherwise indicated

All 19 drivers met inclusion criteria for accelerometer analyses; data were collected for an average of four workdays and two non-workdays at each measurement time point. There were no significant differences in mean work wear time at baseline (10.7 [SD 2.1] hours), end intervention (10.9 [SD 2.0] hours) and follow-up (11.5 [SD 1.7] hours). Over a 24-h monitoring period at baseline, accelerometer data from the random forest classifier indicated that drivers were significantly more sedentary on non-work (14.7 [SD 2.1] hours/day) compared to workdays (10.3 [SD 1.5] hours/day; *p* = 0.000); no differences were observed in time spent physically active on these days (non-work and workday = 1.7 [0.6] hours/day).

Table 3 presents the proportions of time at baseline, end-program and follow-up, spent in physical activity, sedentary and stationary + categories, during work time, and non-work time on work days (24 h minus work time) and non-work days (24 h). There were non-significant increases in mean physical activity times for each of these work and non-work periods at end-program, and at follow-up for work time (+1%; ≈ 7 mins/day). For secondary outcomes, there were non-significant decreases in the mean proportions of work time drivers spent sedentary at end-program (−6%) and follow-up (−9%), with the majority of this time distributed to stationary + (≈40–60 min/day). The opposite was the case for time outside of work, where mean sedentary (inclusive of sleep) and stationary + proportions increased and decreased respectively (e.g. non-work time on workdays at follow-up; 9% ≈ 68 mins/day; *p* = 0.007).

**Table 3:** Accelerometer data (proportions of time spent in physical activity, sedentary and stationary + categories; *n* = 19) and workday dietary choices (*n* = 17) at baseline, end-program and follow-up

	Baseline	End-program	Follow-up
Accelerometers (%)			
Work time			
Physical activity	9 (3)	10 (5)	10 (5)
Sedentary	23 (16)	17 (6)	14 (5)
Stationary+	68 (16)	73 (7)	76 (7)
Workday non-work time (24 h – work time)			
Physical activity	5 (3)	6 (3)	5 (3)
Sedentary^1^	63 (15)	68 (9)	72 (8)
Stationary +^2^	32 (13)	26 (7)	23 (7)
Non-workday (24 h)			
Physical activity	7 (2)	8 (3)	7 (3)
Sedentary	62 (8)	65 (9)	66 (9)
Stationary+	31 (7)	29 (8)	29 (8)
Workday dietary scores			
Mean (SD) fruit serves/day^3^	4 (1)	5 (1)	4 (1)
2+ fruit serves/day (%)	100	100	100
Mean (SD) veg. Serves/day^4^	4 (2)	5 (2)	4 (2)
6+ veg serves/day (%)	29	41	24
Mean (SD) saturated fat score (0–90)	50 (9)	56 (12)	56 (13)
Mean (SD) processed sugar score (0–69)	42 (7)	43 (6)	43 (7)

*SD* standard deviation

Workday non-work time: ^1^Sedentary baseline vs follow-up (*p* = 0.007); ^2^Stationary + baseline vs end-program (*p* = 0.037) and follow-up (*p* < 0.033)

Fruit and veg serves/day: Baseline vs end-program^3^ (*p* = 0.023) and ^4^(*p* = 0.024)

Workday dietary scores are also shown in Table 3. Mean self-reported fruit (*p* = 0.023) and vegetable (*p* = 0.024) consumption significantly increased by one serve/day at end-program, but returned to baseline values at follow-up. Higher food index scores showed a sustained but non-significant reduction in high saturated fat food/beverage choices at end-program and follow-up, while the scores for processed, refined sugar food/beverage choices remained similar across measurement points.

**Fig. 1:**
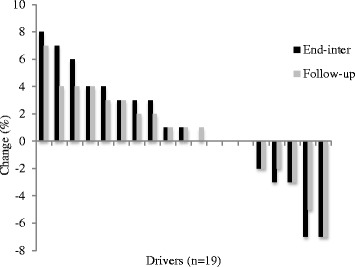
Work time physical activity percentages

**Fig. 2:**
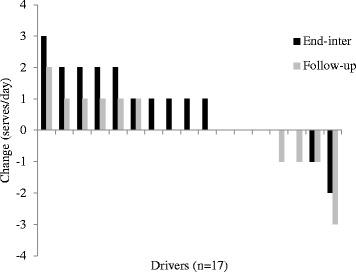
Fruit intake

**Fig. 3:**
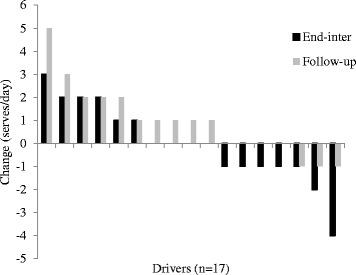
Vegetable intake

**Fig. 4:**
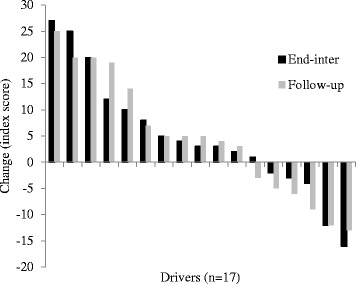
Saturated fat intake

**Fig. 5:**
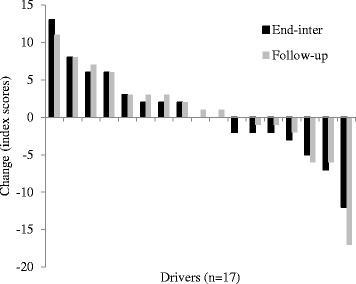
Processed sugar intake

Figures 1, 2, 3, 4 and 5 show the distributions of changes (positive [increase]; 0 [no change]; negative [decrease]) in physical activity and dietary choices at end-program and follow-up for individual drivers; each pair of bars denotes the end-program and follow-up change in a single driver. Changes in work time physical activity varied considerably from driver-to-driver, with percentages (Fig. 1) ranging from an increase of +8% to a decrease of −7% at follow-up (≈50 mins/day). Ten (53%) and 9 (47%) drivers increased the proportion of their daily work time spent physically active at end-program and follow-up respectively.

Variations were also apparent for changes in fruit (Fig. 2; ±3 serves/day) and vegetable (Fig. 3; +5 to −4 serves/day) consumption, and saturated fat (Fig. 4; index score of +25 to −13) and processed, refined sugar (Fig. 5: index score of +13 to −17) food/beverage choices. Most drivers made two or three positive changes from these four dietary categories at end-program (14 drivers [83%]) and follow-up (13 drivers [76%]). A total of nine (53%) and 11 (65%) drivers demonstrated positive changes in at least one workday dietary category and work time physical activity respectively, whereas only one driver demonstrated no positive changes in any dietary category and physical activity at follow-up.

Thematic analyses of end-program interviews (*n* = 17) highlighted that drivers experienced a complex and broad range of barriers that limited physical activity and healthy dietary choices. Perceived lack of time, fatigue and poor physical infrastructure were commonly cited problems. As two drivers described:

*“I know all the things I need to do* (to be healthy) *but I’m also in a difficult position because of this particular job. I do a lot of interstate, often nine days straight. Literally, catching an hour sleep here or there. You are in so much of a hurry to meet deadlines.”*

Long haul driver (Participant 3)

*“You work long hours and you need to make the money. I do think that sleep is a problem and also access to good food. Where do you stop where you can find proper nutritious food? To have more healthy options out there would be excellent”.*

Local delivery driver (Participant 8)

Depot manager comments provided different insights into healthy choice barriers, highlighting modern work practices and policies that have removed the requirement for ‘on the job’ physical activity, as well as a work culture where drivers take on multiple shifts to maintain high salaries and meet financial obligations

*“Today, drivers are way more unfit than 10 or 20 years ago because of the removal of manual physical tasks through smart engineering. Another change is the financial point of view. Being on decent money, they* (the drivers) *spend more. They don’t have to do so many hours but they choose to do it.”*

Depot manager

All interviewees commented on the positive impact the intervention had on encouraging physical activity and health dietary choices. As the following quotes suggest:

*“It was very helpful. What I tried to do was to fit as much movement as I could into the day. While someone was unloading the truck, I was walking with them, or while I was waiting, I’d fit in extra steps. Just by doing that three times a day, I was doing an extra 4,000 steps, which is quite good.”*

Local delivery driver (Participant 11)

*“It kept me accountable to exercise, to food, what I eat and drink”.*

Local delivery driver (Participant 16)

Support by drivers via social media, and the ability to self-monitor and regulate healthy choices were viewed by drivers as valuable facilitators of change. For example:

*“It was nice that when you see* (via the smartphone application) *one of your mates eating something wrong, you say ‘hey, what are you doing?’ It’s nice in that sense that you kind of look after one another.”*

Local delivery driver (Participant 7)

*“As we get older, it’s hard to keep your fitness levels up, so by wearing the band and looking at the data, I could see what I was doing and I knew what to try and improve.”*

Local delivery driver (Participant 16)

However, the physical activity component of the program was considered easier to implement than dietary changes. Drivers commented that monitoring and self-regulating energy intake required high levels of time investment, and that individualised dietary programs and support would have added to the program. As the following quote illustrates:

*A dietician would give you a better insight into what you should be eating and what you shouldn’t and when, and for those who would want to lose some weight, it would be a good choice.*

Local delivery driver (Participant 12)

Drivers valued the competitive element to behaviour change introduced to the program through the accumulation of points relative to small changes. As one driver mentioned:

*It was a good motivator seeing the other driver’s points and comparing them with yours.*

Local delivery driver (Participant 6)

However, consensus from interviews indicated that improving health and wellbeing for its own sake, rather than for external reward, was the major determinant of change. The monetary value of the incentive (which was not significant by driver standards) may also have played a role in this dynamic. As another driver commented:

*“The biggest reward is getting the result we want at the end, and it is to be a healthy driver. To achieve that, we don’t need $100 or $200 here and there.”*

Long haul driver (Participant 2)

Consistent with these viewpoints, descriptive data for the financial incentives aspect of the program indicated that the majority of drivers (*n* = 13; 68%) only accumulated between 50 and 99 lifestyle points and progressed no further than *Gear Shift 2* ($50 reward). Two drivers (11%) achieved *Gear Shifts 3&4* respectively (100–199 points; $80–120 reward), while four drivers (21%) surpassed the maximum threshold of 200 points and achieved *Gear Shift 5* ($200 reward).

In regard to future directions, drivers highlighted the value of *Shifting Gears* as an effective means of promoting awareness of the need for healthy changes, and the flow-on effects this may have for benefiting productivity. As two drivers commented:

*“If your workers are more healthy, there is a good chance they’ll work for you and that increases productivity, and they* (drivers) *will take less time off. I never thought about it until you came to tell us.”*

Local delivery driver (Participant 11)

*“This type of program is what we need for truck drivers. All trucking companies should take a look at their drivers and try to enhance their health and fitness to give a better performance.”*

Local delivery driver (Participant 4)

Lastly, management comments on ‘moving forward’ suggested that choices to effect and sustain healthy changes were up to drivers, while driver viewpoints tended to contrast with management perspectives. For example:

*“It is not so much the organisation’s responsibility it’s the individual’s. We can only do so much.*

Depot manager

*“We would be more driven to try and achieve more if something came from the industry and they said ‘we are going to invest in this way’ to give you more time on the run. But the industry is so ‘hard-nosed’. The bottom line is dollars.”*

Long haul driver (Participant 3)

This last quote reflects the consensus put forward by drivers that the industry needed to take more responsibility for the health of drivers. These responsibilities revolved around better management of shift time and the integrated provision of healthy lifestyle approaches into paid time.

## Discussion

The aim of this study was to assess the impact of a small changes, m-Health financial incentives program on the work time physical activity and workday healthy dietary choices of Australian truck drivers. At 20 weeks and 2 months follow-up, over half of the drivers in our sample demonstrated positive changes in work time physical activity, and an aspect of their workday healthy dietary choices, which included improvements in either fruit, vegetable, saturated fat or refined, processed sugar intake. These data provide encouraging evidence on the feasibility of the *Shifting Gears* program. Importantly, they also highlight that despite significant time and environmental barriers, it is possible to incorporate physical activity and good dietary choices into an occupational routine often considered manifestly unsuited to healthy behaviours.

Drivers commented on the value of using the *Jawbone UP* to track and implement healthy choices in real-time. m-Health interventions that utilise smartphone applications and self-monitoring technology have been proposed as an effective means of empowering hard-to-reach and socially isolated groups (such as truck drivers) towards behaviour change [19]. Our findings add to this growing consensus, with the activity tracker and smartphone application we provided seeming to be the principal mechanism for effecting behaviour change in those drivers who demonstrated increases in physical activity and improvements in diet. However, reflecting our previously reported process data on decreases in the number of smartphone dietary logs made over the intervention timeline, [9] a factor that in all likelihood impacted the number and variability of change in healthy dietary choices, drivers clearly found the knowledge, time and effort required to monitor and self-regulate food and beverages to be more considerable than that needed for physical activity choices. This was due in large part to the functionality of the *Jawbone UP* and the more automated feedback processes inherent in using the activity tracker and download capacity of the smartphone application. Other researchers have identified ‘ease of use’ to be a key aspect of men’s engagement with information technology health interventions, [20] and in line with the feedback we received from drivers, a recent review specific to dietary monitoring has advocated the importance of complementing smartphone-based interventions with input and individualised guidance from experts [21]. ‘Culturally tailoring’ applications to target groups, such as those with low health literacy has also been recognised as important, [22] and this latter aspect may be essential for future initiatives and studies that seek to develop, test and translate m-Health interventions that reflect the unique contexts and challenges of truck driving.

We also sought to facilitate small changes in physical activity and healthy dietary choices by combining self-monitoring with a financial incentives program that rewarded the accumulation of points and small changes overtime. It has been suggested that the provision of external rewards in the form of financial incentives promotes short-term (up to 6 months) engagement with health behaviour interventions [23]. However, recent systematic reviews for physical activity [24] and diet [25] both highlight that the type and size of the reward must be matched to the expectations of the target population if behaviour change potential is to be realised. Our qualitative data suggest that the financial incentive we provided in the form of vouchers with a maximum monetary value of $200 was not significant enough to encourage behaviour change in and of itself. That said, and consistent with the masculinity-based literature that highlights the motivational appeal men place on competition in group-based chronic disease prevention programs, [26] drivers valued and enjoyed contesting and comparing their points progression from month to month. This in turn stimulated social interaction, friendly rivalry and constructive peer support around healthy choices.

Some drivers demonstrated bigger positive changes than others. The largest average improvements at follow-up equated to an extra 45 min of physical activity during an 11-h shift, and an increase of five vegetable serves/day. These findings highlight the potential for integrating healthy choices into truck driver routines. As an example, interview data suggested physical activity increases were accrued through incidental walking while waiting for deliveries to be loaded or unloaded. Equally, it is important to recognise that some drivers demonstrated no, or negative changes in either target behaviour, and for whom the program was ineffective. This may have been due to the need for a more personalised, expert-led intervention, combined with the fact that we did not pursue or fully engage company support for program strategies. Comprehensive ecological approaches to occupational health promotion interventions that target the individual and organisational domains of the work system are more likely to be successful at encouraging changes in lifestyle behaviours than interventions that target any one domain alone [27]. For feasibility testing, we purposely focused on driver aptitude for change. Our next phase of testing will seek to investigate how additional support from other key stakeholders (e.g. unions, companies, regulatory bodies and service station providers) might extend the appeal, reach and scalability of the program to a wider spectrum of drivers, and particularly those less ready or able to implement change.

As secondary outcomes of interest, it was worth noting that the majority of change in sedentary work time at end-program and follow-up proportioned to time spent in stationary + rather than physical activity. From one perspective, these data infer that the program may have successfully facilitated decreases in sitting and increases in standing time during driving breaks. Such an outcome is of interest because of established links between high levels of occupational sedentary exposure and chronic disease outcomes, allied with the metabolic health benefits of interrupting prolonged periods of sitting with standing [28]. However, it is important to recognise that we did not directly measure posture. Furthermore, stationary + also captured driving, and we cannot be sure that time spent driving remained constant over our three measurement periods. Additionally, consideration of sedentary time changes implies the possibility of an intervention ‘compensation’ effect. Cross-sectional Australian data has found no evidence that less physically demanding occupations result in more leisure time physical activity or vice versa [29, 30]. Our data suggest that in this sample of drivers, reductions in sedentary exposure at work might have led to increases in this behaviour outside of work, particularly on a working day. More research on this potential phenomenon is required, but if this is the case, interventions that target drivers must be holistic in nature and consider both work and non-work contexts.

The study had a number of strengths including a multi-method approach with follow-up at 2 months, use of accelerometers and a random forest activity classifier to objectively measure work time sedentary behaviour and physical activity changes, and a comprehensive self-report of multiple dietary components specific to the workday context. The classifier was unable to isolate sleep time, and it will be valuable for future studies to assess intervention impact on sleep duration (and quality), given that long driving hours, shift work and time pressures have been recognised as key factors impacting sleep deficit, fatigue and road accidents in truck drivers [31]. We also recognise the main study limitations of an uncontrolled pre-post design and a small sample size for feasibility testing, which compromised external validity and detection of statistically significant changes in target behaviours. Lastly, our study experiences highlight that recruitment of long-haul drivers into future intervention studies will be challenging. Until larger studies are undertaken with representative groups, applicability of our program to different types of drivers must be considered with caution, given that our sample was biased towards those employed in the local delivery industry.

## Conclusions

This is the first study to investigate the application of smartphone technology and financial incentives for behaviour change in truck drivers, or indeed any other blue-collar occupational group. The findings add to the very limited intervention evidence base, and highlight the potential benefits of using an m-Health approach to encourage drivers and other blue-collar workers to monitor and self-regulate small positive changes in physical activity and healthy dietary choices. However, relative to the size and type of reward provided, our feasibility data also indicated that the financial incentives aspect of the *Shifting Gears* program was not an effective enabler of behaviour change, even though drivers tended to enjoy and engage with the competitive element of accumulating small changes and points across 20 weeks. Additionally, the program was ineffective for some drivers who, particularly for food and beverage choices, may have required the additional support and guidance of a dietician. Ongoing testing of the program now needs to occur with industry stakeholder involvement and larger, representative groups of drivers using a comparative group design.
